# XTIP – the world’s first beamline dedicated to the synchrotron X-ray scanning tunneling microscopy technique

**DOI:** 10.1107/S1600577520003689

**Published:** 2020-04-14

**Authors:** Volker Rose, Nozomi Shirato, Michael Bartlein, Alex Deriy, Tolulope Ajayi, Daniel Rosenmann, Saw-Wai Hla, Mike Fisher, Ruben Reininger

**Affiliations:** a Advanced Photon Source, Argonne National Laboratory, 9700 S Cass Ave, Lemont, IL 60439, USA; b Center for Nanoscale Materials, Argonne National Laboratory, 9700 S Cass Ave, Lemont, IL 60439, USA

**Keywords:** synchrotron X-ray scanning tunneling microscopy, circularly polarizing undulator beamlines, soft X-rays

## Abstract

In recent years, there have been numerous efforts worldwide to develop the synchrotron X-ray scanning tunneling microscopy (SX-STM) technique. Here, the inauguration of XTIP, the world’s first beamline fully dedicated to SX-STM, is reported. The XTIP beamline is located at Sector 4 of the Advanced Photon Source at Argonne National Laboratory. It features an insertion device that can provide left- or right-circular as well as horizontal- and vertical-linear polarization. XTIP delivers monochromatic soft X-rays of between 400 and 1900 eV focused into an environmental enclosure that houses the endstation instrument. This article discusses the beamline system design and its performance.

## Abstract 2

A new beamline, XTIP, has been constructed at the Advanced Photon Source to deliver monochromatic soft X-rays of between 400 and 1900 eV for the emerging synchrotron X-ray scanning tunneling microscopy technique.

## 1. Introduction   

For more than three decades, scanning probe microscopes have been an indispensable tool for the direct high-resolution study of surfaces. Scanning tunneling microscopy (STM) can resolve surfaces with atomic resolution but direct elemental determination of materials is not easily accomplished. X-ray microscopies, on the other hand, provide elemental and magnetic selectivity but currently have a limited spatial resolution. Nevertheless, the strength of X-rays resides in their ability to excite core electrons of a specific level by tuning the incident photon energy to their binding energy. Furthermore, quantitative information about magnetic moments can be obtained because X-ray absorption by a magnetic material depends on the helicity of the incident X-rays. The emerging synchrotron X-ray scanning tunneling microscopy (SX-STM) technique combines the high spatial resolution of STM with the information obtained through X-ray/matter interaction. The technique takes advantage of the fact that X-ray excited electrons can modulate the tunneling current in STM leading to structural, elemental, electronic, and magnetic contrast with high spatial resolution (Saito *et al.*, 2006 ▸; Chiu *et al.*, 2008 ▸; Okuda *et al.*, 2009 ▸; Cummings *et al.*, 2012 ▸; Onderwaater *et al.*, 2016 ▸). A specialized smart tip in close proximity to the sample surface serves as a detector for the X-ray enhanced tunneling current (Rose *et al.*, 2011 ▸; Cummings *et al.*, 2017 ▸). The tip height of typically less than 1 nm is controlled by an advanced feedback loop (Wang *et al.*, 2013 ▸). Elemental sensitivity down to the limit of single atomic height (Shirato *et al.*, 2014 ▸; Kersell *et al.*, 2017 ▸) as well as magnetic contrast (Rose *et al.*, 2012 ▸; DiLullo *et al.*, 2016 ▸) have been demonstrated.

The XTIP beamline is the first beamline worldwide fully dedicated to SX-STM. Previously, substantial effort had been dedicated toward the development of the technique at other synchrotron facilities such as the ESRF (Comin, 2007 ▸). So far, only one related, but conceptually different, beamline combining X-ray absorption and scanning force microscopy currently operates at the Swiss Light Source (Pilet *et al.*, 2012 ▸). The XTIP beamline uses a circularly polarizing undulator (CPU) that can provide left- or right-circular as well as horizontal- and vertical-linear polarization states. The beamline is capable of operating at soft X-ray energies from 400 to 1900 eV and can provide a focused beam down to ∼10 µm × 10 µm into the SX-STM endstation instrument. The spatial resolution in SX-STM is foremost governed by the properties of the tip detector and not the X-ray beam spot size on the sample. Therefore, the goal of XTIP is to provide high flux density with variable resolving power (λ/Δλ) at a given wavelength λ into an X-ray spot that is large enough to illuminate the entire surface area that is usually imaged in SX-STM (typically in the nanometer square to few-micrometer square range). A temperature-controlled and soundproofed enclosure has been constructed in order to provide a stable and low-vibration environment for the endstation instrument.

## 2. X-ray optical layout   

**Figure 1:**
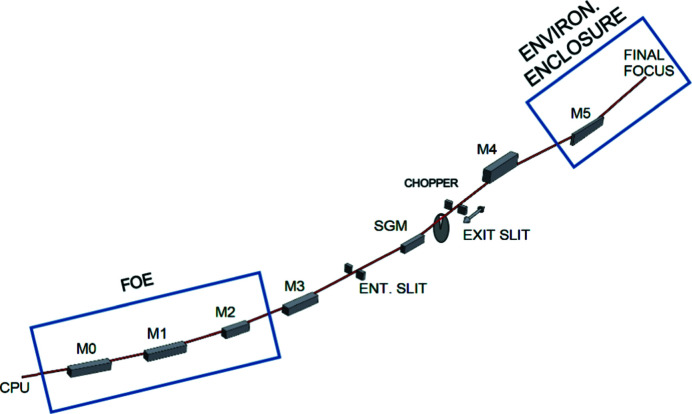
Schematic of the XTIP beamline optical layout.

The XTIP beamline is located at 4-ID of the Advanced Photon Source. Besides a single optical element positioned after the exit slit, the beamline uses horizontally deflecting grazing-incidence elements, which allow a monochromator to accept the entire vertically coherent portion of the beam. The XTIP branch shares the CPU source with the 4-ID-C branch. The CPU is 2.4 m long and features a period of 12.8 cm with 35 vertical and 36 horizontal poles. Additionally, Sector 4 includes a hard X-ray branch (4-ID-D) with the standard Advanced Photon Source undulator A (Freeland *et al.*, 2002 ▸). The canted orientation of the hard X-ray undulator and the CPU provides a 400 µrad splitting of the two beams. Fig. 1 ▸ shows a schematic of the optical path of the XTIP beamline. The first two mirrors in the optical path, M0 (plane) and M1 (spherical), are used by both the C branch and XTIP. Also located in the first optical enclosure (FOE) is M2, a horizontally deflecting plane mirror, which when inserted into the beam serves to deflect the soft X-ray beam into the XTIP branch. When the mirror is retracted, the beam proceeds unaltered down the C-branch optical path. A horizontally focusing cylindrical mirror, M3, focuses the beam onto the entrance slit of the spherical grating monochromator (SGM), which in turn focuses the diffracted beam onto a moveable exit slit. An optical chopper located between the SGM and the exit slit allows modulation of the X-ray beam. A pair of mirrors is used to focus the beam vertically and horizontally (M4 and M5, respectively) at the sample position to a 10 µm × 10 µm-sized spot. The M5 mirror and the endstation instrument are located inside of the environmental enclosure.

## 3. Beamline physical layout   

**Figure 2:**
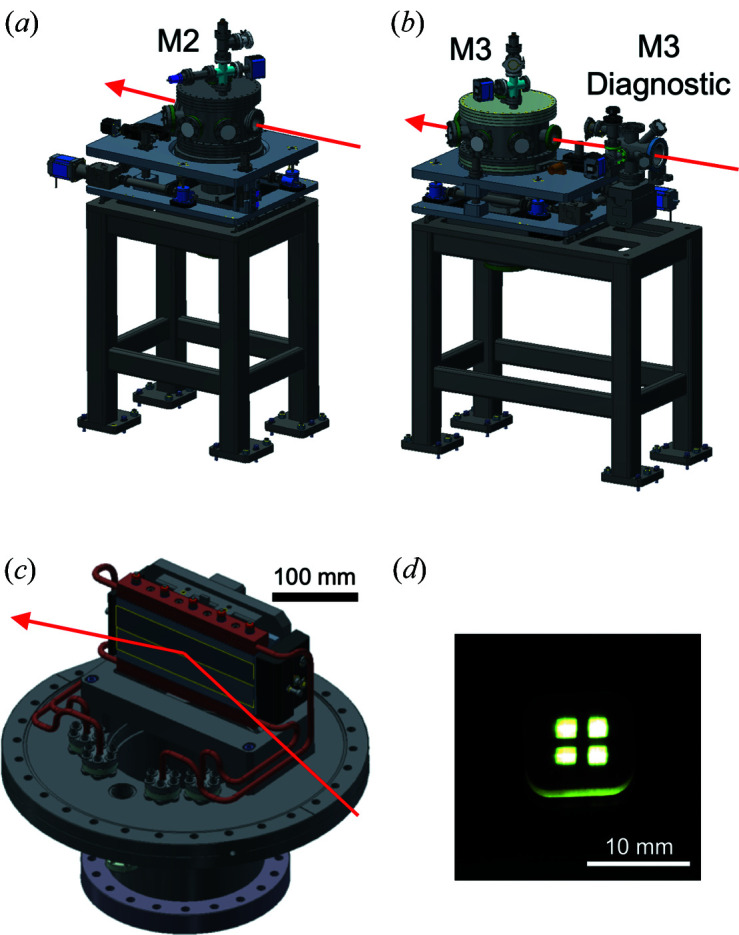
(*a*) The M2 mirror assemblies pass the beam either into XTIP or the C branch at 4-ID. (*b*) The steel frame of M3 includes a diagnostics assembly. (*c*) The mirror mount of M2 and M3 exhibits a modular design allowing for the mounting of the optics into its holder followed by the mounting and strain relief of the cooling block. (*d*) The beam projected on the YAG screen inside of the M3 diagnostic assembly, with the horizontal and vertical wire probes in the beam path.

The physical layout of the XTIP beamline is summarized in Table 1 ▸. The shared mirrors M0 [distance from source (dfs): 30.23 m] and M1 (dfs: 32.65 m) are described elsewhere (Freeland *et al.*, 2002 ▸). Fig. 2 ▸(*a*) shows the side-bounce mirror assembly of the M2 mirror (dfs: 33.65 m), which deflects the beam horizontally, when inserted into the beam, away from the 4-ID-C branch and into XTIP. The assembly provides a vertical travel of ±25 mm to allow for the selection of one of two optical mirror coatings, carbon or platinum. The carbon coating can be used for harmonic rejection of the undulator beam and has higher reflectivity at lower photon energies of between 400 and 1000 eV. Fig. 2 ▸(*b*) displays the M3 assembly (dfs: 39.15 m) that focuses the beam horizontally onto the SGM entrance slit. The M3 mirror is a platinum-coated meridional cylinder with a radius of 311.2 m. Both M2 and M3 utilize a modular mirror holder [Fig. 2 ▸(*c*)]. The mirror mount design features six fixed contact points with six corresponding spring-loaded contacts. The cooling blocks clamp around the water tubing and are spring loaded against the side of the mirror. The mirror mount also acts as a baffle to shield the mirror surface from an ion pump that is mounted at the bottom of the vacuum chamber. In addition, a glow discharge screen is situated at the opening to the ion pumps to protect the mirror from ion-pump discharges. On both M2 and M3 there are upstream masks to protect the mirror surfaces and electrically isolated downstream masks that are used as a diagnostic when aligning the beam.

**Figure 3:**
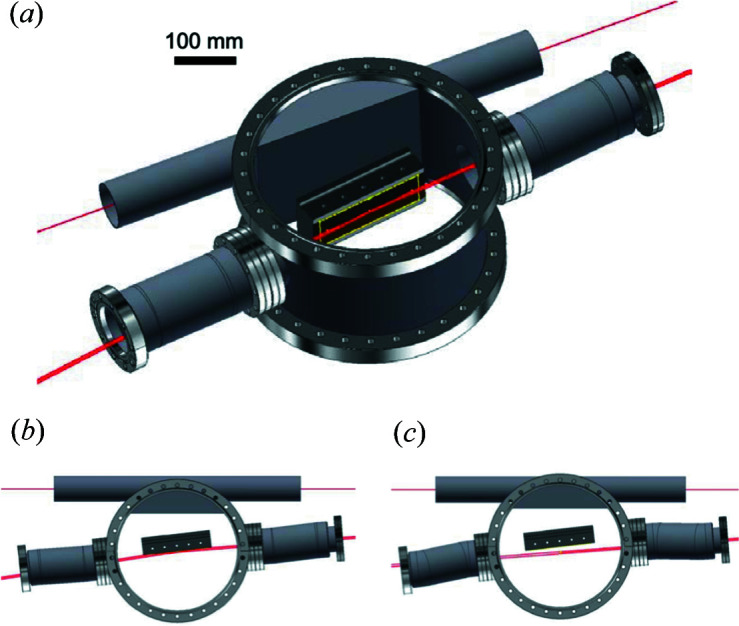
(*a*) A schematic of the M2 assembly, with the beam traveling from right to left. The hard X-ray beam of the D branch passes through the beam pipe shown above M2. (*b*) The soft X-ray beam is deflected into XTIP. (*c*) With retracted mirror, the soft X-ray beam passes into the C branch.

Fig. 3 ▸ depicts a schematic of the M2 assembly. When M2 is inserted, it deflects the beam into the XTIP branch [Fig. 3 ▸(*b*)]. When M2 is retracted, the beam passes into the C branch [Fig. 3 ▸(*c*)]. The hard X-ray beam of the D branch passes through a separate beam pipe outside of the M2 assembly.

**Figure 4:**
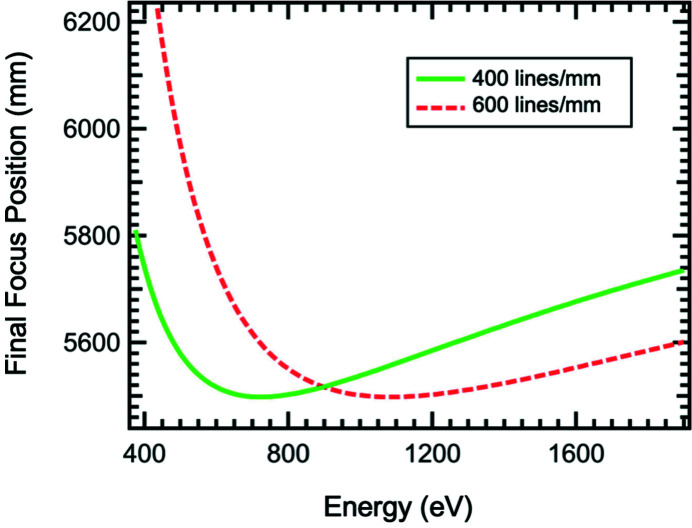
SGM focus location as a function of photon energy for the 400 and 600 lines mm^−1^ gratings. The exit slit is designed to track the focus about the nominal focal (exit-arm) distance of 5.5 m from the grating.

The monochromator is a horizontal diffracting SGM (Chen, 1987 ▸). It has an included angle of 175.5°, a 3.5 m entrance arm and a nominal 5.5 m exit arm (McNulty *et al.*, 1996 ▸). The water-cooled entrance slit (dfs: 42.65 m) is located at a fixed position upstream of the SGM (dfs: 46.15 m). The SGM is operated in the first inside order. The diffracted beam is focused on a movable exit slit adjustable over a 660 mm range (dfs: 52.0 ± 0.33 m). Each of the slits has a phospho­rus-coated mask upstream of the slit blades which can be used for steering the beam. The SGM focus location as a function of energy is shown in Fig. 4 ▸. The grating chamber houses two holographically recorded ion-beam etched gratings with 114.6 m curvature radius and line densities of 400 and 600 lines mm^−1^. The 400 lines mm^−1^ grating with 25 nm-thick Pt coating is optimized at ∼800 eV. The 600 lines mm^−1^ grating, previously used at beamline 2-ID-B of the Advanced Photon Source (McNulty *et al.*, 1997 ▸), is coated with 20 nm Rh and exhibits the highest photon flux at ∼1100 eV allowing extension of the operation to higher energies. The gratings are contact cooled with copper foils with each optic being supported in kinematic holders with six adjustment axes. The SX-STM technique requires modulation of the X-ray beam. Therefore, an ultra-high-vacuum compatible optical chopper (dfs: 50.5 m) operating at frequencies of up to 6 kHz is added to the beam path (Chang *et al.*, 2016 ▸).

**Figure 5:**
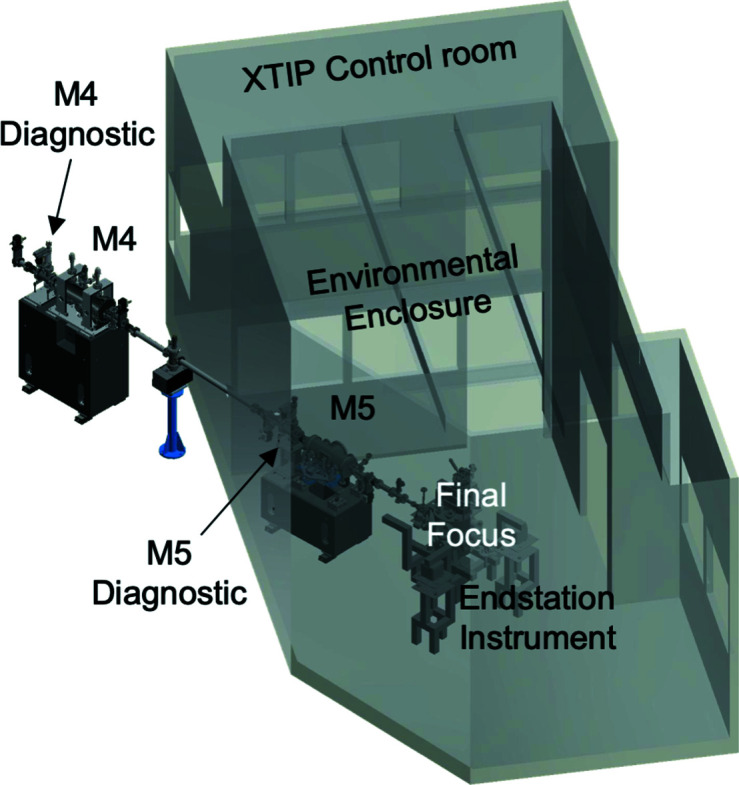
The vertically deflecting focusing mirror M4 and bendable horizontally deflecting focusing mirror M5 provide a final focus of ∼10 µm × 10 µm at the location of the endstation instrument. Both mirror assemblies include a diagnostic chamber. An environmental enclosure provides a temperature-controlled and noise-proof setting for the endstation instrument. The beamline is operated from the adjacent control room.

The final focus of ∼10 µm × 10 µm is achieved with a Kirkpatrick–Baez mirror pair, M4 and M5 (Fig. 5 ▸). Both mirrors are uncooled and have a platinum coating. The vertical focusing mirror, M4 (dfs: 54.0 m), is a meridional elliptical cylinder and is the only vertically deflecting element in the beamline. It images the source, originating from the center of the CPU, onto the sample position. Since the exit slit has 660 mm of total travel, M5 (dfs: 58.0 m) is a bendable mirror focusing the horizontal exit slit onto the sample. The final focus is located 60.0 m away from the source, at the position of the endstation instrument. The environmental enclosure enhances the temperature stability and reduces noise at the final-focus position. This is achieved through wall panels with acoustical-type solid fill as well as by means of double-pane glass windows. A supply air diffuser is used to minimize air-flow disturbances. The experiments are carried out remotely from inside the adjacent XTIP control room.

An effort was made to place a diagnostic before each component in the optical path. As such, there are diagnostic chambers before M3, the SGM, M4 and M5. These diagnostic chambers include a gold-coated tungsten mesh with 12.5 µm wire diameter on a 64 µm grid, a Ce-doped YAG crystal and, after the monochromator, a photodiode. The M5 diagnostic further houses standard foils for energy calibration. In addition to the diagnostics mentioned above, horizontal and vertical electrically isolated tungsten wire probes are located between M2 and M3. The wire probes can be independently scanned through the beam. This is used to characterize the position, size and intensity of the beam. The vertical wire is also used to determine the relative position of the straight through beam (C branch) and the deflected beam (XTIP) to allow fast and easy switching between the two branches. In addition to knowing the beam profile at the wire location, the wire can be used to cast a shadow down the optical path aiding in the beamline alignment. An image of the wire probes projected on the YAG screen of the M3 diagnostic is shown in Fig. 2 ▸(*d*).

## 4. Beamline performance   

**Figure 6:**
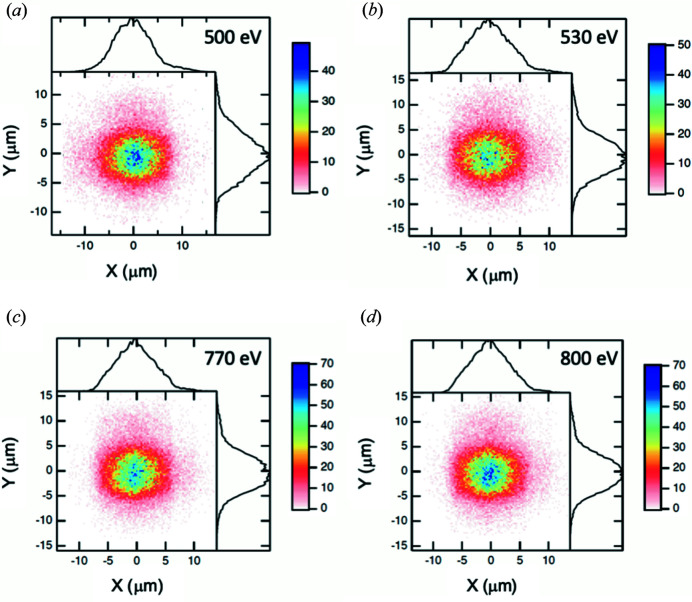
Calculated beam profiles at the sample including slope errors on all optical components. The spot size and position are preserved within 30 eV when the exit slit is fixed. Beam profile for (*a*) 500 eV and (*b*) 530 eV (exit slit: 23 µm fixed at the 530 eV location; entrance slit: 27 µm). The spot size remains at 9 µm × 9 µm FWHM. Likewise, for a photon energy of (*c*) 770 eV and (*d*) 800 eV (exit slit: 23 µm fixed at 800 eV; entrance slit: 27 µm), a spot size of 8 µm × 8 µm FWHM is preserved.

The goal of XTIP is to deliver a monochromatic X-ray beam of ∼10 µm × 10 µm with selectable polarization and high positional stability to the final-focus location. Since its horizontal source is the moving exit slit, special emphasis was devoted to the accuracy and reproducibility of the exit-slit movement. Additionally, in order to guarantee ultimate positional stability of the final-focus position, calculations were carried out to evaluate potential focal-spot broadening for small-range energy scans (30 eV), in which the exit-slit position remains fixed. This energy range covers, for example, spectroscopy scans over the *L*_2_ and *L*_3_ absorption edges of transition metals. Fig. 6 ▸ shows the calculated beam profile at the final focus for photon energies of 500, 530, 770 and 800 eV. These shadow ray-tracing calculations (*SHADOW* hybrid) (Shi *et al.*, 2014 ▸) include diffraction effects from optics apertures and slope errors as listed in Table 1 ▸. In the case of 500 and 530 eV the FWHM beam size changes from 8.7 µm × 8.5 µm to 8.5 µm × 8.5 µm. Likewise, for 770 and 800 eV the beam profile changes from 8.1 µm × 8.2 µm to 8.1 µm × 8.1 µm, respectively. Obviously, the spot size is preserved for small-range energy scans demonstrating that the exit-slit location can be fixed if the highest positional stability of the final focal is desired.

**Figure 7:**
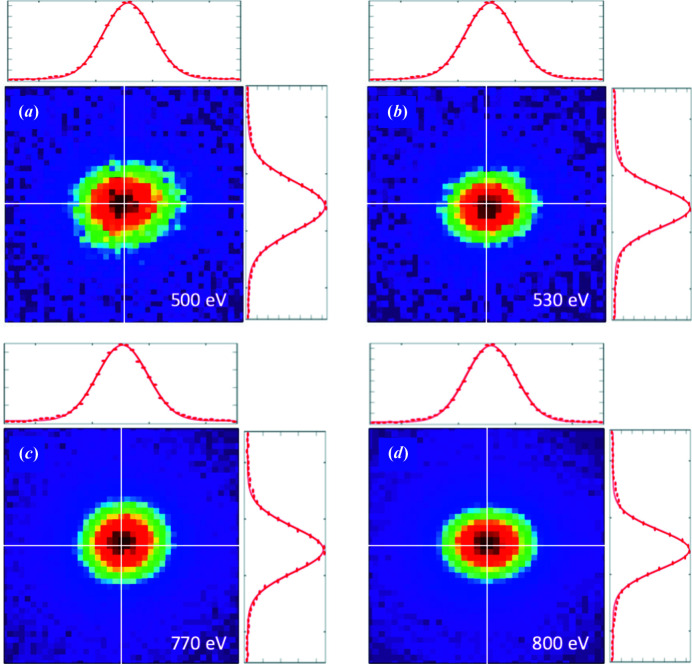
Final focus measured using a YAG crystal. The total field of view shown is 40 µm × 40 µm. The exit-slit size was 25 µm for all images and the exit-slit position was fixed at 530 eV and 800 eV.

The calculated beam stability has been verified experimentally. Fig. 7 ▸ shows the beam profile measured at the final focus using a YAG crystal and a high-resolution CCD camera. The final-focus position is very well preserved for small-range energy scans as demonstrated for energy ramps from 500 to 530 eV and 770 to 800 eV using fixed exit-slit positions at the 530 eV and 800 eV SGM focus, respectively. Similar to the final-focus position, the beam spot size is also well preserved. The final focus has been fit to a Gaussian distribution. With an exit-slit size of 25 µm, the measured spot sizes are 12 µm × 10 µm (500 eV), 11 µm × 9 µm (530 eV), 11 µm × 10 µm (770 eV) and 10 µm × 9 µm (800 eV) FWHM. Hence, the experimental spot sizes agree well with the theoretical prediction.

**Figure 8:**
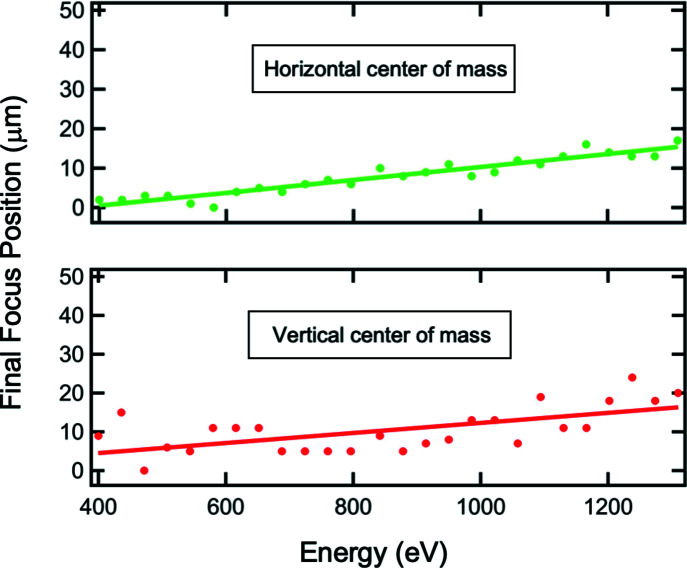
Shift of the center of mass of the final-focus position in the horizontal and vertical directions as a function of photon energy. The exit-slit location is adjusted to track the SGM focus and M5 is bent to maintain the smallest focus. However, the positional degrees of freedom of the M4 (vertical) and M5 (horizontal) focusing mirrors are fixed, which can be used to correct for the shift.

For wider energy scans, the position of the exit slit has to be adjusted to track the focus of the SGM (*c.f.* Fig. 4 ▸). Fig. 8 ▸ shows the shift of the center of mass at the final-focus position as a function of energy using the 400 lines mm^−1^ grating. The curvature of the horizontal-focusing mirror is adjusted to maintain the smallest spot size for each energy. However, the positional degrees of freedom of the M4 and M5 focusing mirrors are not utilized. In the horizontal and vertical directions, the final focus continuously shifts over ∼15 µm upwards and 15 µm outwards for the scanned energy range 400–1300 eV. The slightly higher noise observed in the vertical direction can be attributed to insufficient stiffness of the mount supporting the high-resolution camera imaging the beam on the YAG screen. Because it is important in SX-STM to always illuminate the junction between the sample and the tip, a shift larger than half of the final-focus size needs to be corrected. The positional degrees of freedom of the M4 and M5 mirrors can be utilized for that purpose. Alternatively, the microscope inside of the SX-STM endstation instrument can be adjusted to follow the final-focus location.

**Figure 9:**
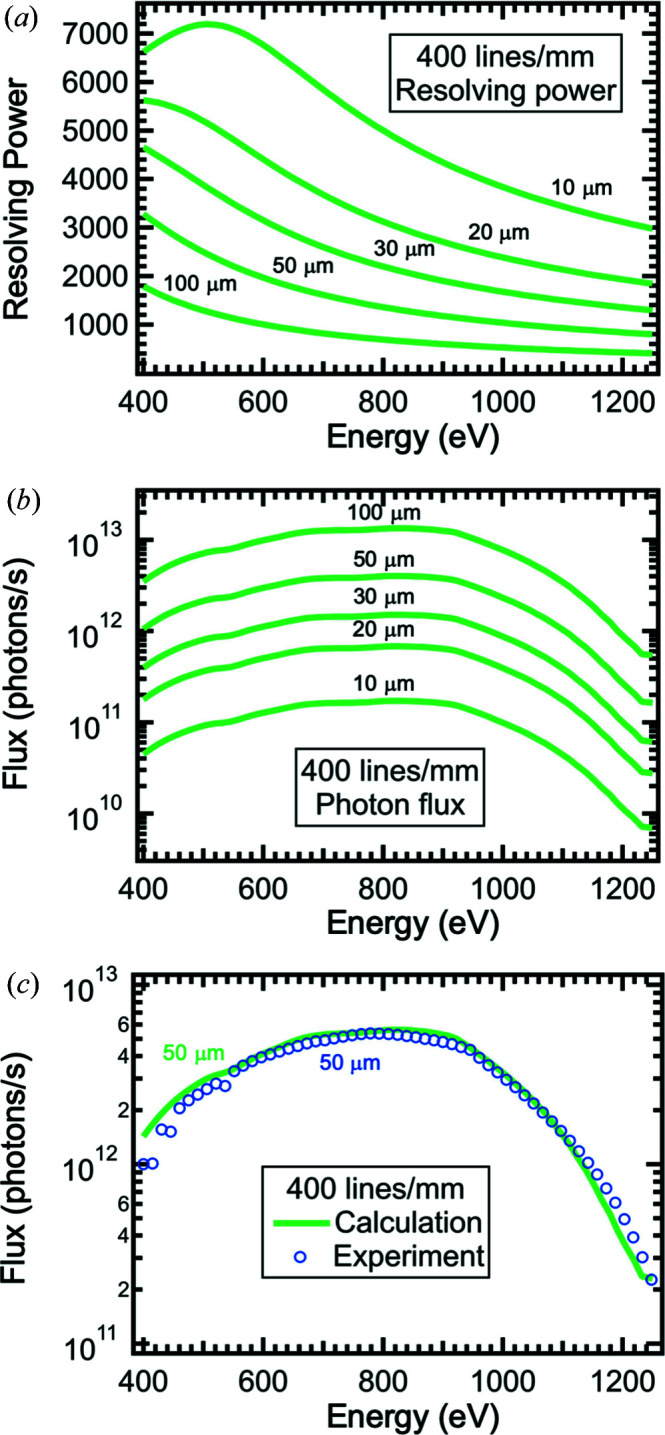
Data for the 400 lines mm^−1^ grating. (*a*) Calculated resolving power of the XTIP beamline. The resolving power is shown for slit sizes of 10, 20, 30, 50 and 100 µm. The exit slit and entrance slit are set to the same value. (*b*) Respective calculated photon flux at the final-focus position. (*c*) Comparison of the calculated photon flux with the experimentally obtained photon flux at the M5 diagnostic chamber for 50 µm slits.

The calculated resolving power λ/Δλ and the photon flux of the 400 lines mm^−1^ grating at the final-focus position as a function of photon energy are shown in Fig. 9 ▸. In the calculations, the flux emitted by the insertion device, the reflectivity of all mirrors and the grating efficiency are taken into account. The entrance- and exit-slit sizes are set to the same value and calculations for 10, 20, 30, 50 and 100 µm are presented. In the case of 10 µm entrance and exit slits, a resolving power of up to ∼7200 at ∼520 eV can be achieved [Fig. 9 ▸(*a*)]. The resolving power then decreases with increasing slit size. The flux expected at the final-focus position as a function of photon energy is shown in Fig. 9 ▸(*b*). As can been seen in the figure, the flux covers multiple orders of magnitude, typically from 10^10^ to 10^13^ photons s^−1^, depending on the entrance- and exit-slit settings. In contrast to the resolving power, the photon flux increases with increasing slit size. The photon flux is experimentally evaluated using a calibrated photodiode located in the M5 diagnostic assembly [Fig. 9 ▸(*c*)]. The measured photon flux is in good agreement with the expected flux at the M5 diagnostics as demonstrated for the example of 50 µm entrance and exit slits. The experimental photon flux reaches ∼2 × 10^12^ photons s^−1^ at 800 eV.

**Figure 10:**
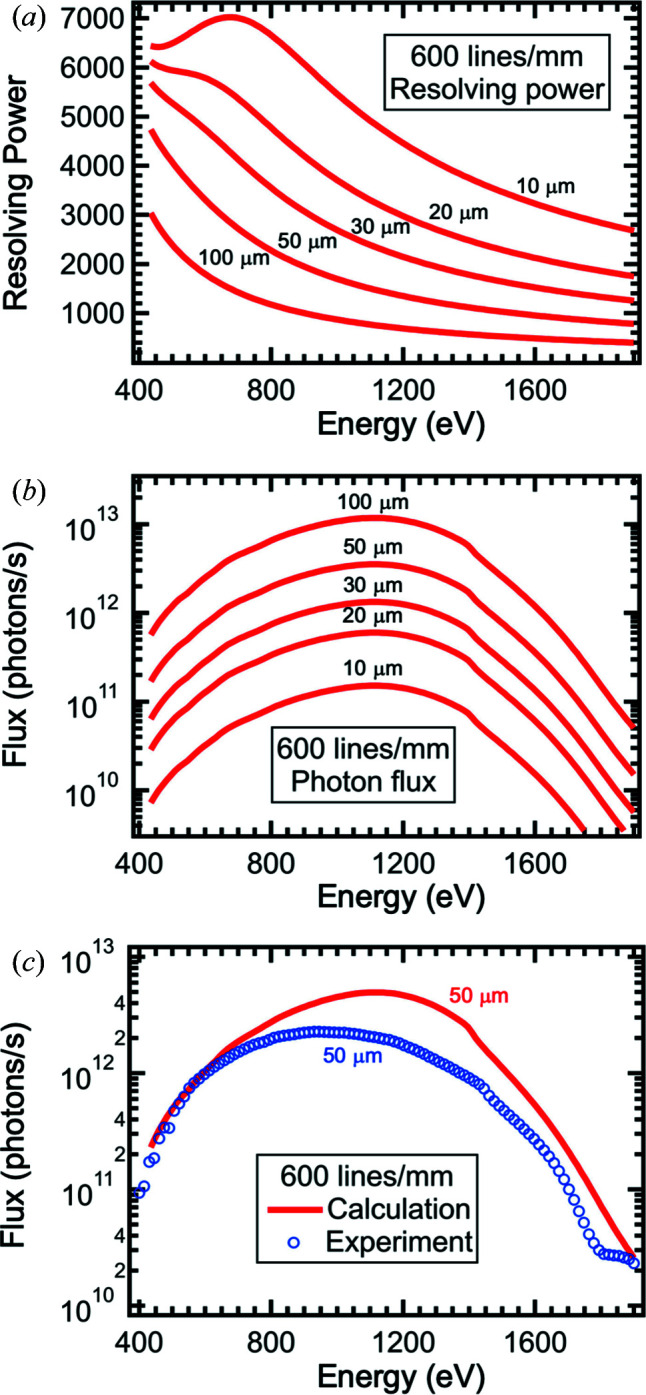
Data for the 600 lines mm^−1^ grating. (*a*) Calculated resolving power at the final-focus position. (*b*) Calculated photon flux at the final-focus position. (*c*) Comparison of the calculated photon flux with the experimentally obtained photon flux at the M5 diagnostic chamber for 50 µm entrance and exit slits.

Fig. 10 ▸ shows the calculated resolving power and the photon flux of the 600 lines mm^−1^ grating at the final-focus position. Generally, in comparison with the 400 lines mm^−1^ grating, higher groove densities, *i.e.* more lines per millimetre, deliver higher reciprocal dispersion and therefore higher resolution for the same slit settings. However, the coma error starts to dominate the resolution at energies below ∼600 eV. Therefore, a maximal resolving power of 7020, similar to the 400 lines mm^−1^ grating, is obtained, but it is shifted to a higher photon energy of ∼670 eV [Fig. 10 ▸(*a*)]. Likewise, the highest photon flux of ∼10^11^ to 10^13^ photons s^−1^ is shifted to ∼1100 eV [Fig. 10 ▸(*b*)]. The 600 lines mm^−1^ grating slightly underperforms in peak flux compared with the expectation, as shown in Fig. 10 ▸(*c*). In the case of 50 µm slits, at the M5 diagnostic chamber a flux of ∼2 × 10^12^ photons s^−1^ is observed experimentally at 1100 eV. At the same time, the maximum is shifted to a lower energy than expected. The measured flux peaks with 2.3 × 10^12^ photons s^−1^ at ∼940 eV indicating that the grating does not entirely conform with the specifications listed in the work of McNulty *et al.* (1997 ▸). Therefore, to enhance the photon flux at higher energies, a new high-quality 600 lines mm^−1^ grating is being prepared using new substrates polished together with the current high-quality 400 lines mm^−1^ grating and will be installed in the near future.

**Figure 11:**
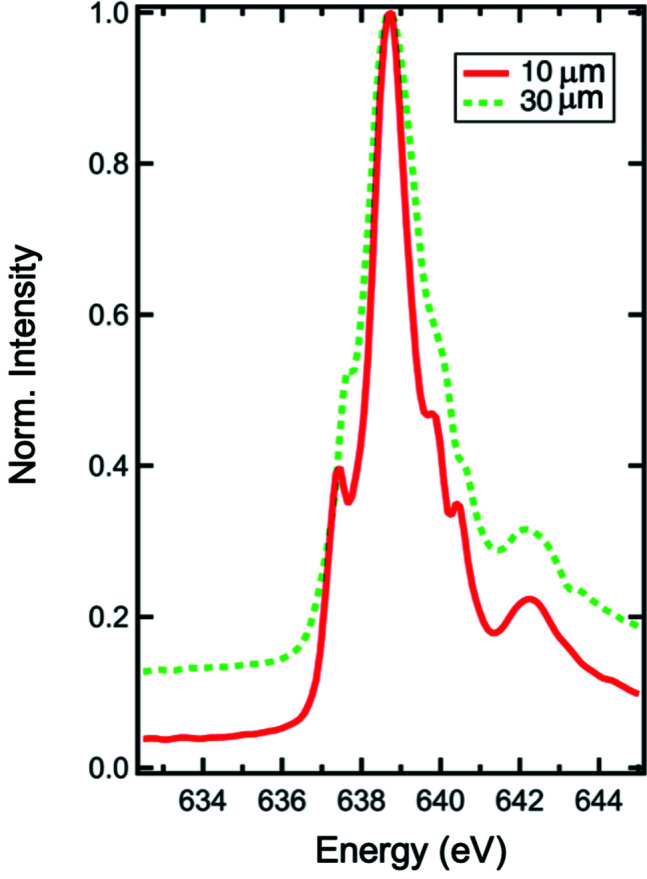
Spectra of an Mn standard foil located in the M5 diagnostic chamber obtained with 10 and 30 µm exit-slit sizes and a fixed 20 µm entrance slit.

The influence of selecting a specific resolving power, *i.e.* energy resolution, can be seen in Fig. 11 ▸. It shows spectra of an oxidized Mn metal calibration standard with an area density of 59.6 µg cm^−2^ ± 5% located in the M5 diagnostics. The spectra were taken with 10 µm and 30 µm slit sizes using the 400 lines mm^−1^ grating. Besides the main Mn *L*_3_ peak at 638.7 eV, there are additional peaks observed at 637.4, 639.8, 640.4 and 642.3 eV. These additional peaks can be assigned to the three oxidation states of manganese, namely M^2+^, M^3+^, and M^4+^ (Anjum *et al.*, 2011 ▸; Khan *et al.*, 2014 ▸). Obviously, the oxidation-state peaks are much more clearly resolved in the case of a 10 µm exit slit (λ/Δλ ≃ 6400) compared with a 30 µm exit slit (λ/Δλ ≃ 2900). The entrance slit is fixed at 20 µm. Likewise, the FWHM of the main Mn *L*_3_ peak decreases from 2.0 to 1.3 eV when using the smaller exit-slit size. It should be noted that the natural width of absorption peaks in solid-state samples is normally larger than the calculated energy resolution of the beamline. A direct experimental evaluation of the energy resolution would therefore require noble-gas resonances. Generally, the resolving power increases with reduced slit sizes. However, at the same time the photon flux decreases. In practice, by selecting one of the two gratings, the energy, photon flux and resolving power can be tailored for a specific experiment. Usually, imaging experiments (Shirato *et al.*, 2014 ▸) at XTIP require high photon flux, while spectroscopy measurements (Chang *et al.*, 2018 ▸) benefit from high energy resolution.

**Figure 12:**
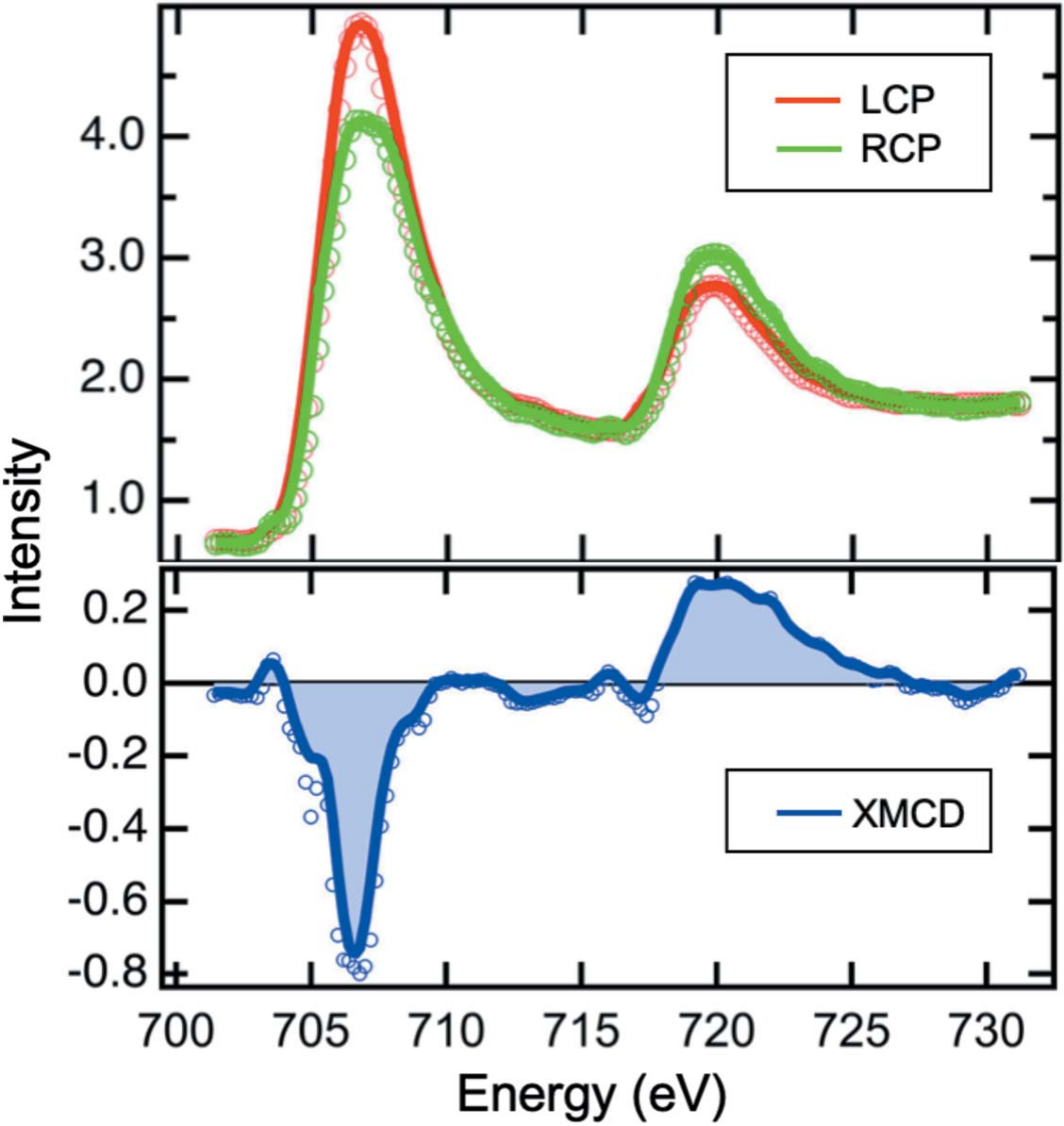
X-ray absorption spectra of an iron thin film measured at the final-focus position (top). The spectra taken with LCP and RCP X-rays exhibit different intensities of the Fe *L*_3_ and Fe *L*_2_ absorption edges. The XMCD spectrum (bottom), derived from the difference of the two absorption spectra, provides access to the magnetic properties of the thin film.

The XTIP beamline utilizes a CPU with full polarization control providing the ability to study magnetic properties of materials. Fig. 12 ▸ shows absorption spectra of an iron thin film measured with left-circular polarized (LCP) and right-circular polarized (RCP) X-rays. The Fe film with a thickness of ∼50 monolayers was deposited on a Au(111) substrate and post-annealed at 740 K for 90 min. The sample preparation and measurements were carried out under ultra-high vacuum conditions. For the measurement, the sample was kept at 90 K and illuminated by a focused soft X-ray beam at a grazing angle of ∼5°. The spectra measured using a specialized smart tip exhibit peaks at 706.8 and 719.9 eV, which are associated with the Fe *L*_3_ and Fe *L*_2_ absorption edges, respectively. The relative peak intensities change with the X-ray polarization because the X-ray absorption of a magnetic sample is spin dependent. In this case, the X-ray absorption proceeds from the 2*p* core shell, involving the excitation of electrons from the spin-orbit split 3*p*_3/2_ (Fe *L*_3_) and 3*p*_1/2_ (Fe *L*_2_) levels into the 3*d* shell, whose incomplete occupation is the origin of a magnetic moment. The magnetic moments of the Fe film can then in principle be obtained by recording an X-ray magnetic dichroism (XMCD) spectrum, derived from the differences in the absorption intensities of X-rays with right- and left-circular polarization (Stöhr, 1995 ▸).

## 5. Conclusions   

A new beamline, XTIP, has been constructed at the Advanced Photon Source to deliver monochromatic soft X-rays between 400 and 1900 eV for the emerging SX-STM technique. The source for this beamline is a CPU, providing full polarization control. A pair of focusing mirrors delivers a focused beam of 10 µm × 10 µm with a variable photon flux in the range of ∼10^12^ to 10^13^ photons s^−1^ with a resolving power of up to ∼7200. The beamline is the world’s first dedicated branch for the SX-STM technique. It will offer the scientific user community unique ways to explore nanoscale materials.

## Figures and Tables

**Table 1:** Overview of the physical layout of the XTIP beamline h: horizontally; v: vertically; def: deflecting; foc: focusing; *p*: source–mirror distance; *q*: mirror–image distance; *R*: radius of curvature.

Optical component	dfs (m)	Angle of incidence (°)	Optical surface (mm)	Shape	Slope errors (µrad)	Description
M0 mirror	30.23	88.9	260 × 60 rhodium	Plane		Side cooled, h def
M1 mirror	32.65	88.9	260 × 60 rhodium	Sphere *R* = 1610 m		Side cooled, h def, very weakly foc
M2 mirror	33.65	87.8	150 × 60 carbon, platinum	Plane	1	Side cooled, h def
M3 mirror	39.15	88.5	220 × 15 platinum	Meridional cylinder *R* = 311.2 m	1	Passively cooled, h def, h foc
Entrance slit	42.65					Water cooled, blades open h
SGM	46.15	Included angle 175.5	170 × 20 400 lines mm^−1^ Pt 600 lines mm^−1^ Rh	Sphere *R* = 114.6 m	0.4 (400) 0.5 (600)	h diffracting
Optical chopper	50.50					Variable frequency
Exit slit	52.00					Blades open h, translates along beam ±330 mm
M4 mirror	54.00	88.5	290 × 15 platinum	Meridional elliptical cylinder *p* = 54 m *q* = 6 m	0.4	v def, v foc
M5 mirror	58.00	88.5	400 × 15 platinum	Plane	1	Bendable, h def, h foc
Final focus	60.00					
